# To What Extent Does Yellow Rust Infestation Affect Remotely Sensed Nitrogen Status?

**DOI:** 10.34133/plantphenomics.0083

**Published:** 2023-09-06

**Authors:** Alexis Carlier, Sebastien Dandrifosse, Benjamin Dumont, Benoît Mercatoris

**Affiliations:** ^1^ Biosystems Dynamics and Exchanges, TERRA Teaching and Research Center, Gembloux Agro-Bio Tech, University of Liège, 5030 Gembloux, Belgium.; ^2^ Plant Sciences, TERRA Teaching and Research Center, Gembloux Agro-Bio Tech, University of Liège, 5030 Gembloux, Belgium.

## Abstract

The utilization of high-throughput in-field phenotyping systems presents new opportunities for evaluating crop stress. However, existing studies have primarily focused on individual stresses, overlooking the fact that crops in field conditions frequently encounter multiple stresses, which can display similar symptoms or interfere with the detection of other stress factors. Therefore, this study aimed to investigate the impact of wheat yellow rust on reflectance measurements and nitrogen status assessment. A multi-sensor mobile platform was utilized to capture RGB and multispectral images throughout a 2-year fertilization-fungicide trial. To identify disease-induced damage, the SegVeg approach, which combines a U-NET architecture and a pixel-wise classifier, was applied to RGB images, generating a mask capable of distinguishing between healthy and damaged areas of the leaves. The observed proportion of damage in the images demonstrated similar effectiveness to visual scoring methods in explaining grain yield. Furthermore, the study discovered that the disease not only affected reflectance through leaf damage but also influenced the reflectance of healthy areas by disrupting the overall nitrogen status of the plants. This emphasizes the importance of incorporating disease impact into reflectance-based decision support tools to account for its effects on spectral data. This effect was successfully mitigated by employing the NDRE vegetation index calculated exclusively from the healthy portions of the leaves or by incorporating the proportion of damage into the model. However, these findings also highlight the necessity for further research specifically addressing the challenges presented by multiple stresses in crop phenotyping.

## Graphical abstract

**Figure:**
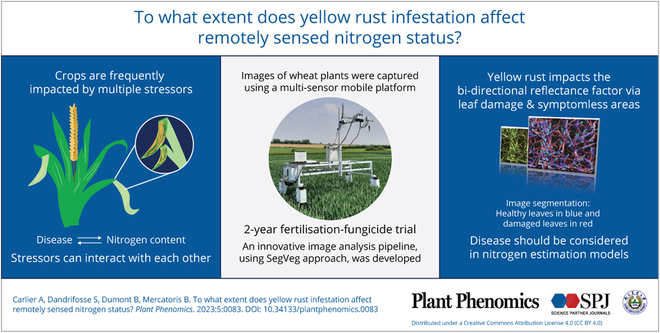

## Introduction

In field conditions, crops are exposed to several stresses at the same time. Whether biotic, such as pests and diseases, or abiotic, such as drought and nutrient deficiency, stresses result in the reduction of the quantity and/or the quality of the harvest. While agricultural inputs have historically been the primary means of mitigating these stresses on a large scale, their extensive use has recently been the subject of many societal and environmental concerns. Moreover, the emergence of concurrent or sequential stresses can exacerbate their negative impact, altering the pattern of symptoms and further hindering crop productivity and stress identification [1,2]. On this basis, understanding how plants respond to multiple stresses is essential for improving crop yield and quality [2].

Stress identification and quantification have become common practices using remotely sensed data. Recent plant phenotyping methods offer new possibilities to screen plants in high-throughput, nondestructive, and objective way [3,4]. They have been identified as promising tools to assist plant improvement [5–7]. Nonetheless, the diversity of data acquisition systems, data management, and analyses bring many challenges to the phenotyping community [8–10].

In this context, the investigation of abiotic stresses continues to be a prominent and ongoing subject of study [11]. One particular area of focus is the detection of nitrogen deficiency, which plays a crucial role in agricultural practices, particularly in relation to fertilization strategies [12,13]. Insufficient nitrogen availability in crops can result in reduced biomass growth and the manifestation of yellowing leaves, both of which are key indicators of nitrogen deficiency. Spectral data analysis, facilitated by techniques such as machine learning, radiative transfer modeling, and vegetation indices (VIs), either independently or in combination, has proven to be effective in tracking these symptoms across the entire crop areas [13–16]. Moreover, it is important to acknowledge that the presence of background elements, such as soil, can introduce mixing and disturbances to the targeted data associated with the specific plant under study. For instance, Song et al. [15] reported that the accuracy of nitrogen status estimation using a spectroradiometer was relatively lower during the early growth stage (GS) of crops compared to the subsequent vegetative phase. Consequently, certain researchers have effectively addressed this issue of spectral mixing at the canopy level. Wang et al. [17] proposed a novel approach known as abundance-adjusted VIs, which mitigates spectral mixing at the canopy level and enhances the accuracy of leaf nitrogen concentration estimation. Noteworthy achievements have been attained by researchers who have focused their analysis on specific regions within the canopy. For instance, Jay et al. [18] successfully estimated beet chlorophyll content by concentrating on the most illuminated pixels of green vegetation.

Under field conditions, the detection of diseases still comes up against many difficulties such as the similarity of symptoms, the possibility of observing them, and the diversity of plant responses [19]. Common methods include the use of spectral data [20] or RGB images [21]. For instance, Anderegg et al. [22] has quantified Septoria tritici blotch (STB) using spectral and temporal features from a spectroradiometer. Other image analysis methods, such as textural analysis from proximal multispectral images, were relevant to estimate the severity of wheat main diseases [23]. Recently, deep learning algorithms are paving a new avenue for plant phenotyping [24–26]. In particular, convolutional neural networks (CNNs) have indeed demonstrated their good performances in phenotyping task related to object detection [27], segmentation [28], or disease classification [29,30].

Despite all these stress detection possibilities, very few studies have addressed the effects of multi-stress and their interactions on single trait estimation, as depicted by Berger et al. [19] and Zhang et al. [31]. The authors emphasized the need for further research to address this challenge. In fact, all mentioned studies related to nitrogen stress estimation have treated the question in optimal management practices, while it is known that diseases could substantially disturb the nitrogen dynamic [32]. The nutritional habit of the disease could induce a reduction in leaf nitrogen concentration [33], and thus induce a bias in the nitrogen status estimation. Hyperspectral systems appear as the most suitable sensor to face this challenge. For instance, Devadas et al. [34] was able to delimit stripe rust and nitrogen deficiency using specific VIs with a spectrometer. Another solution might be the use of multi-sensor approach. For instance, while symptoms associated with a pathogen were similar to those of water stress, Zarco-Tejada et al. [35] were able to distinguish between both stresses using the combination of hyperspectral and thermal sensors. Generally speaking, many biotic stresses manifest visible symptoms that can be segmented provided sufficient spatial resolution. In this context, close-range systems such as mobile platforms or gantries are good candidates, as they can carry multiple sensors in close range and thus provide high-resolution data [36].

This paper presents an approach to investigate the impact of yellow rust (YR) on VIs that are usually used in the frame of nitrogen status retrieval. The hypothesis is that the diseases induce a bias in the estimation of nitrogen status not only through its visible symptoms but also through its biological interaction with the plant. To test this hypothesis, proximal RGB and multispectral images were acquired on a wheat field trial spanning 2 cropping seasons, where different fungicide applications and nitrogen inputs were combined. The methodology involved isolating the leaves within the images, segmenting disease symptoms (i.e., leaf damages), and using the resulting mask to study the correlation between VIs from healthy or diseased leaves, and nitrogen status variables of the plant.

## Materials and Methods

### Field experiments

A winter wheat trial was conducted during the cropping seasons 2020–2021 and 2021–2022 in Lonzée, Belgium (50°33′50″N and 4°42′00″E). The pedoclimatic context is characterized by a deep silt loamy soil and a temperate climate. In the trial, 15 treatment combinations that involved varying levels of nitrogen inputs and fungicide applications were completely randomized. These combinations can be viewed in Table 1. The total nitrogen inputs were split into 3 applications, during tillering phase (GS 23 to 25), at stem elongation (GS 30), and at flag leaf (GS 39) stages. GSs are related to the BBCH-scale [37]. The fertilization scenarios were designed to test various levels of nitrogen input, ranging from the traditional recommendation of 3 inputs of 60 kgN ha^−1^ to an excess of 260 kgN ha^−1^, a deficiency of 120 kgN ha^−1^, and a level designed to promote tillering 200 kgN ha^−1^. Ammonium nitrate (27%) was used. Several strategies of fungicide applications were conducted: no fungicide application (0F); a single fungicide application during the flag leaf stage GS 39 (1F); 2 fungicide applications at the second node GS 32 and heading GS 55 stages (2F); and 3 fungicide applications at GS 32, GS 39, and grain development (GS 70) stages (3F). Fungicide mixtures were composed of triazole (Kerstrel, 1.25 L ha^−1^) at GS 30, triazoles-pyrazoles-carboxamides (Librax, 1.5 L ha^−1^) at GS 39 and 55, and triazole (Prosaro, 1 L ha^−1^) at GS 70. The susceptible cultivar “LG Vertikal” was sown on 2020 October 20 with a density of 275 seeds m^−2^, and the very susceptible cultivar “Bennington” was sown on 2021 October 28 with a density of 300 seeds m^−2^. For both cropping seasons, the previous crop was potato. The experimental plots measured 1.95 m × 6 m, and the inter-row spacing was 0.14 m. Each treatment had 4 replicates for the data acquisition, which represent 60 plots and 4 more replicates only dedicated to final grain yield measurements using a combine harvester.

Different types of agronomic data were collected on June 2 and 16 for 2021 at GS 39 and 65, respectively, and on April 11, May 2, 17, and June 2, 21 for 2022 at GS 30, 32, 39, 65, and 75, respectively. First, above-ground biomass was sampled in the 3 central rows over 50 cm long. Samples were collected on 3 replicates for the 7 treatments highlighted in bold in Table 1. Fresh wheat plants were manually divided into separate organs, i.e., stems, leaves, and ears. Plant materials were dried into an oven at 65 °C until constant weight. Dry organs were then weighted. Their nitrogen concentration (%N) was determined using the Dumas method. The nitrogen uptake (Nuptake) in kgN ha^−1^ was calculated by multiplying the dry matter and the corresponding nitrogen concentration. The nitrogen nutrition index (NNI) was computed using the traditional approach from Justes [38]. All these reference measurements are summarized as nitrogen status variables in the rest of this paper. Finally, grain yields were obtained on the 4 other replicates for all treatments. Unfortunately, violent storms affected the trial in 2021 and many plots have lodged several weeks before harvest. Thus, 2021 grain yield will not be studied in this research.

The 3 main leaf diseases, namely, STB, YR, and brown rust (BR), were graded on field according to the visual score (VS) scale commonly used by the regional experts, for all treatments (Table 2). The grade is based on the average intensity of the disease on the highest affected foliar floor and for a certain number of plants. It is a fast scoring method that represents the global incidence of the disease of the plot. It was rescaled following Eq. 1 so that a scaled visual score (sVS) equal to 0 means no disease and 1 corresponds to a very high level of disease pressure. In 2021, due to a low disease pressure, scoring has been performed only on 4 dates, on June 16, 25, and July 2, 9. In 2022, diseases appeared early during the season; thus, the trials were scored 10 times on April 19, 25, May 2, 9, 17, 23, 23, 30, and June 2, 13, 21.

$$\mathrm{sVS}=\frac{9-\mathrm{VS}}{8}$$ (1)

**Table 1.** Trial treatments that involve combinations of both the total nitrogen input and the number of fungicide applications. Treatment combinations in bold were dedicated to dynamic destructive data sampling.

Nitrogen input (kgN ha^−1^)	Number of fungicide application (F)			
	0F	1F	2F	3F
120	**120_0F**	**120_1F**	120_2F	**120_3F**
180	180_0F	**180_1F**	180_2F	180_3F
200	200_0F	200_1F	/	200_3F
260	**260_0F**	**260_1F**	260_2F	**260_3F**

**Table 2.**
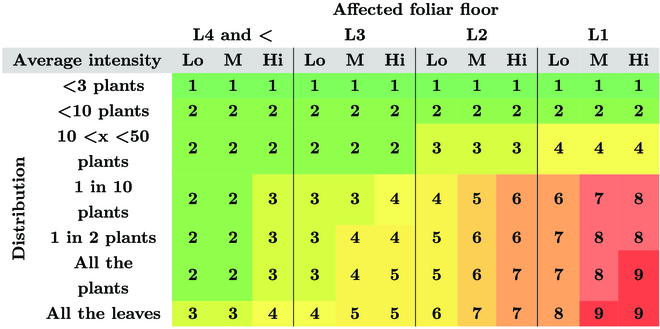
Scale for the visual scoring of wheat fungal diseases. The scale is based on 3 criteria: (a) the affected foliar floor (L1 refers to the flag leaf, L2 to the second upper leaf, …), (b) the average intensity of the infection on a leaf (Lo = low, M = medium, and Hi = high), and (c) the distribution of the disease in the plot or within the plant leaves.

**Table 3.** IoU and accuracy of the SegVeg model. Soil-Plant results refer to the U-NET model, and the Green-Damage results refer to the pixel-wise classifier.

		Soil-Plant		Green-Damage	
		EfficienNetB2	ResNet34	XGBoost	SVM
Training	IoU	0.89	0.84	0.85	0.86
	Accuracy	0.94	0.91	0.92	0.92
Validation	IoU	0.76	0.67	0.78	0.79
	Accuracy	0.87	0.83	0.88	0.88

**Table 4.** Rate of change between BRFs of image and leaves, and between leaves and green elements in %.

	BRF 490		BRF 550		BRF 680		BRF 720		BRF 800	
Image-Leaves	Leaves-Green	Image-Leaves	Leaves-Green	Image-Leaves	Leaves-Green	Image-Leaves	Leaves-Green	Image-Leaves	Leaves-Green
2022-GS30	0.40	−1.70	16.28	−0.70	−23.30	−3.22	32.42	−0.49	41.81	0.35
2022-GS32	−1.08	−1.47	9.55	−2.332	−28.00	−3.60	19.87	−1.81	26.60	−0.29
2022-GS39	20.83	−1.36	20.15	−3.444	−15.37	−10.56	22.23	−2.57	31.04	1.08
2022-GS65	20.15	−3.77	23.17	−5.77	4.47	−12.53	25.08	−4.89	26.94	−0.68
2022-GS73	11.72	−2.744	6.74	−3.06	10.15	−9.63	8.72	−2.67	16.59	0.51

**Table 5.** *P* values (ANOVA) of NNI, %N leaves, Nuptake leaves, %N total, and Nuptake total at different growth stages, where “total” refers to the entire plant.

Source of variation	2021-GS39	2021-GS65	2021-GS89	2022-GS30	2022-GS32	2022-GS39	2022-GS65	2022-GS75	2022-GS89
NNI									
Fertilization (N)	<0.01	<0.01	<0.01	0.08	<0.01	<0.01	<0.01	0.197	<0.05
Fungicide (F)	0.199	0.531	0.429	0.404	0.965	0.414	0.676	0.055	<0.05
N × F	0.814	0.198	0.553	0.328	0.579	0.914	0.058	0.185	0.473
%N leaves									
Fertilization (N)	<0.01	<0.01	<0.01	<0.01	<0.01	<0.01	<0.01	<0.05	<0.01
Fungicide (F)	0.55	0.456	<0.01	0.96	0.815	<0.05	<0.01	<0.01	<0.01
N × F	0.845	<0.05	0.642	0.355	0.263	0.679	0.056	0.169	0.535
Nuptake leaves									
Fertilization (N)	0.27	<0.01	<0.01	0.562	<0.01	<0.01	<0.01	<0.01	<0.01
Fungicide (F)	0.392	0.631	0.074	0.156	0.904	0.225	<0.05	<0.01	<0.01
N × F	0.445	0.237	0.706	0.451	0.672	0.724	0.285	0.538	0.385
%N total									
Fertilization (N)	<0.01	<0.01	<0.01	<0.01	<0.01	<0.01	<0.01	0.269	<0.01
Fungicide (F)	0.322	0.463	0.313	0.96	0.9	0.461	0.476	0.487	0.342
N × F	0.379	0.098	0.242	0.355	0.383	0.985	0.254	0.179	0.736
Nuptake total									
Fertilization (N)	0.232	<0.01	<0.05	0.562	<0.01	<0.01	<0.01	0.151	<0.05
Fungicide (F)	0.385	0.62	0.36	0.156	0.857	0.467	<0.05	<0.01	<0.01
N × F	0.384	0.349	0.769	0.451	0.722	0.867	0.09	0.238	0.346
NDRE _green_									
Fertilization (N)	<0.01	<0.01		0.409	<0.01	<0.01	<0.01	0.682	
Fungicide (F)	<0.01	<0.05		0.512	0.568	<0.01	<0.01	<0.01	
N × F	0.417	0.891		0.72	0.258	0.248	0.905	0.352	

### Image acquisition

**Fig. 1.**
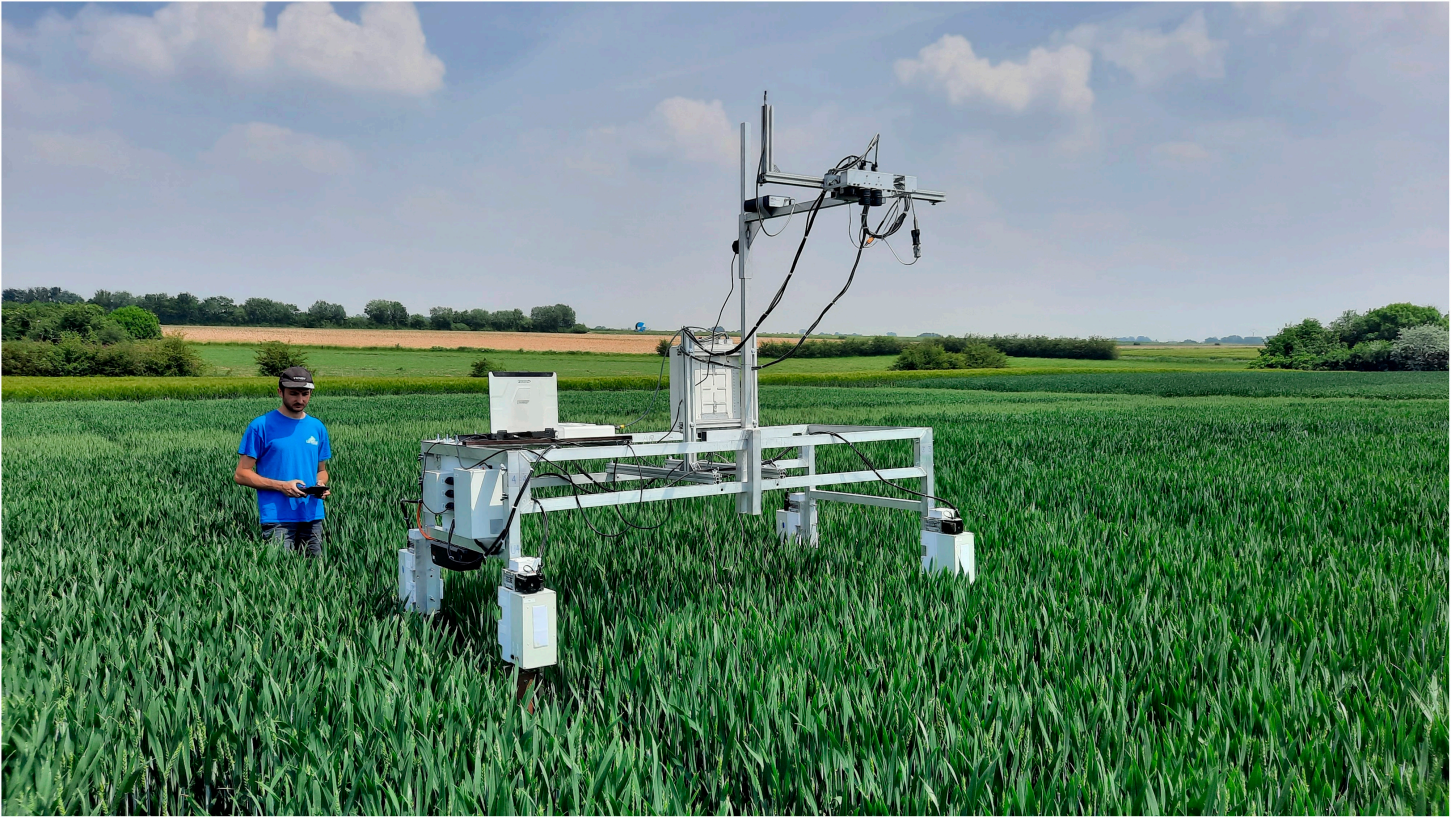
Mobile platform used in this study with the sensor pod fixed 1.6 m above the canopy.

A multi-sensor system was set on a cantilever beam of a mobile platform to acquire nadir images (Fig. 1). It was composed of a multispectral camera array, an incident light spectrometer, and one RGB camera. The multispectral camera array was Micro-MCA (Tetracam Inc., Gainesville, FL, USA) consisting of 6 monochrome cameras equipped with 1,280 × 1,024 pixel complementary metal oxide semiconductor (CMOS) sensor. They were mounted with narrow band-pass optical filters centered at 490, 550, 680, 720, 800, and 900 nm. Each band had a width of 10 nm, except for the 900-nm band, which had a width of 20 nm. The RGB camera was a GO-5000C-USB RGB camera (JAI A/S, Copenhagen, Denmark) with a 2,560 × 2,048 pixel CMOS sensor and an LM16HC objective (Kowa GmbH, Düsseldorf, Germany). Finally, the spectrometer was AvaSpec-ULS2048 equipped with a cosine corrector (Avantes, Apeldoorn, The Netherlands). The trigger of the different sensors was synchronized.

In addition to the images acquired on the same date as the biomass samples, the trials were monitored continuously throughout the cropping season, starting from tillering and continuing until full maturity. A total of 16 acquisition dates was recorded for the year 2021, and 13 dates for the year 2022. Four and 3 image acquisitions per plot were made on all treatments in Table 1 for 2021 and 2022, respectively. To ensure accurate and consistent image acquisition, the camera height was maintained at a constant level throughout each day of data collection. The goal was to position the sensors at a height of approximately 1.6 m above the top of the canopy. This allowed for an observation area greater than 1 m^2^ and optimized the image registration process, as detailed below.

### Color image segmentation

#### SegVeg method

A robust RGB image segmentation technique, known as SegVeg, was utilized in this study, as described in the work by Serouart et al. [39]. To ensure accurate and reliable results, the method employed a combination of 2 distinct techniques: (a) a deep learning approach for soil–vegetation segmentation and (b) a pixel-wise approach for green-yellow vegetation segmentation. This approach allowed for the generation of a robust model that effectively removes soil, enables specific classification of plant pixels, and reduces the effort of annotating them. Furthermore, employing a binary classification approach twice would not result in detrimental instances of misclassification, unlike a 3-class approach where a disease could potentially be misidentified as soil.

The deep learning approach was based on the popular U-NET model, which has been successfully applied in various image segmentation tasks [40]. To leverage the benefits of pretrained models and accelerate training convergence, the U-NET structure utilized different predefined encoders, or backbones, that were pretrained on ImageNet [41]. The encoder, which is the down-sampling part of the U-NET, was implemented using 2 state-of-the-art architectures—ResNet34 proposed by He et al. [42] and EfficientNetB2 proposed by Tan and Le [43]. The decoder, or the up-sampling part of the U-NET, was implemented as a classical design.

The pixel-wise segmentation was carried out using features from the RGB images. Different color spaces and transformations were computed, namely, the normalized RGB channels, the HSV, the CIELab, and the Sobel filter. That made a total of 10 features per pixel. Two models were tested: the support vector machine (SVM) widely used in phenotyping [44], and the eXtreme Gradient Boosting known as XGBoost, a bagging approach known for its performances and rapidity.

The implementation of the algorithms was done using the “Segmentation Models” package by Iakubovskii [45], Tensorflow 2.4, XGBoost 1.7, and Scikit-learn 1.2.

#### Dataset preparation and training

The VegAnn dataset [46] has been enhanced with additional images from the current study. This dataset comprises a collection of RGB images along with corresponding binary masks for plant–soil segmentation. The VegAnn dataset encompasses 3,775 multi-crop RGB images captured under diverse illumination conditions, using various systems and platforms, and representing different phenological stages.

To ensure the adequacy of the model to the present study, 30 RGB images from the 2022 dataset and 8 from the 2021 dataset have been selected. The RGB images have been manually segmented into 2 classes: soil and plant parts with and without damage. The masks were generated using the plug-in Labkit [47] from the Fiji software [48]. It is a user-friendly platform for manual and automated image segmentation. The segmentation process involved manually drawing the soil and plants on a few areas in the image, after which a fast random forest based pixel classifier was used to segment the entire image. The number of manually drawn areas varied depending on the annotator’s judgement and the heterogeneity of the image and comprised several dozens of pixels. This approach allowed for improving the integrated random forest by adding more labels to regions that were poorly predicted by the classifier. Although less accurate than manual labeling, this tool provided a faster way to generate masks.

Then, the images and corresponding masks were partitioned into 20 nonoverlapping images of 512 × 512 pixels, similar to the VegAnn dataset. One-third of this dataset was used as the validation dataset, comprising a total of 260 patches.

For training, a batch size of 16 was set for 100 epochs. The Adam optimizer with default parameters of Tensorflow was used, and each image was scaled according to the corresponding backbone, similar to the ImageNet preprocessing. The dice loss was chosen as the loss function as it presents better capacity to handle unbalanced dataset.

Second, to generate a comprehensive pixel-wise segmentation dataset, 120 RGB images from the 2022 dataset were selected for training, and 33 RGB images from the 2021 dataset were selected for validation. The manual annotations included a few pixels of both green and damaged parts to ensure a balanced distribution of each class. This annotation was also done using the Labkit tool without applying the random forest classifier. The selection of images was based on the need to represent the heterogeneity of images encountered. Thus, the training dataset comprised approximately 15,000 pixels for each class, while the validation dataset contained around 2,500 pixels for each class.

#### Evaluation metrics

Models were evaluated using the accuracy as a standard metric (Eq. 3). A common other metric for semantic segmentation is the intersection over union (IoU) (Eq. 2) with a threshold of 0.5. It is the ratio between the area formed by the overlap of the predicted and the labeled regions and the area formed by the set of these 2 regions. It ranges from 0 to 1. The lower the IoU, the worse the prediction result. The computing configuration was a NVidia Tesla V100 GPUs.

$$\mathrm{IoU}=\frac{\text{Intersection}}{\text{Union}}=\frac{\mathrm{TP}}{\mathrm{FP}+\mathrm{TP}+\mathrm{FN}}$$ (2)

$$\text{Accuracy}=\frac{\mathrm{TP}+\mathrm{TN}}{\mathrm{TP}+\mathrm{FP}+\mathrm{TN}+\mathrm{FN}}$$ (3)

TP, TN, FP, and FN stand for the number of pixels of true positives, true negatives, false positives, and false negatives, respectively.

### Image analysis pipeline

#### Processing of images

**Fig. 2.**
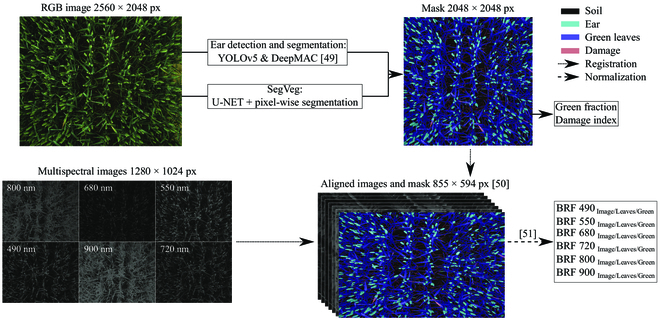
Image analysis pipeline. RGB images were used to segment the scene into soil, ear, green leaf, and damage. Combined with multispectral images, the pipeline allows to extract the BRFs of each mentioned classes.

The developed image analysis pipeline is presented in Fig. 2. First, the RGB image was used to segment the soil, the green plant parts that were mainly leaves, and the damaged parts (see the “Color image segmentation” section). Then, wheat ears were detected using YOLOv5 and segmented within each bounding box by the Deep Mac model according to the procedure proposed by Dandrifosse et al. [49]. Those masks were combined to build a mask with 4 classes: the soil, the ears, the damaged parts of the leaves, and the green parts of the leaves. Note that the leaves refer to the leaves complemented by the stem parts visible in a nadir view. Second, these masks were applied on the multispectral images. Due to the proximity with the canopy and the spatial shift between the different lenses of the cameras, images needed to be aligned in a way that each pixel had the same position across all images. Therefore, image registration was computed following the algorithm from Dandrifosse et al. [50]. It used a B-Spline method to account for local deformations.

Furthermore, the high variability of sunlight conditions encountered was recorded by the incident light spectrometer. Using these data, the multispectral images could be normalized against the illumination conditions. Thus, the method proposed by Dandrifosse et al. [51] was carried out to compute an estimation of bidirectional reflectance factor (BRF), more commonly called reflectance, from which VIs could be calculated. BRFs were also corrected at each date of acquisition using a known reflectance panel before and after the trial acquisition. Consequently, BRF_Image_ denotes BRF for the entire image, BRF_Leaves_ denotes BRF for the entire leaves, and BRF_Green_ represents BRF for the green parts of the leaves.

Five VIs were selected for their demonstrated relationships with crop nitrogen status and plant health, disease, or senescence (Table S1). These VIs included the normalized difference red edge (NDRE) index [52], the modified normalized difference blue index (mDNb) [18], and the chlorophyll index red edge (CIred-edge) [53], which are known to be correlated with crop nitrogen status, and the normalized difference vegetation index (NDVI) [54] and the plant senescence reflectance index (PSRI) [55,56] for their sensitivity to plant health, disease, or senescence.

#### Foliar damage quantification

On the basis of the RGB segmentation, the proportion of pixels representing green and damaged parts of the plants were computed. The green fraction (GF) was defined as the number of green plant pixels divided by all pixels in the image; meanwhile, the damage index (DI) was calculated as the proportion of damage pixels relative to the sum of the green plant and damage pixels. The study made the assumption that the observed damage was primarily attributed to disease and that no other sources of damage, such as physiological or insect-related damages, were observed throughout the experiment.

To correctly assess the disease importance throughout the growing season and take into account the temporal dynamics of the severity of the disease, the area under the disease progression curve (AUDPC) was computed following the procedure proposed by Simón et al. [32]. Thus, AUDPC_sVS_ was calculated from the sVS scores observed within plots, whereas AUDPC_DI_ was computed from DI extracted from the mask.

### Statistical analysis

The study employed a comprehensive data analysis approach that integrated various statistical methods. An analysis of variance (ANOVA) was conducted to assess the impact of treatments on both image features and agronomical data. However, it was important to exclude the 180_1F treatment from the agronomic data analysis. This exclusion was necessary because this specific nitrogen treatment had only one fungicide factor, which could introduce bias into the statistical analysis. Therefore, to ensure accurate interpretation of the results, it is crucial to consider this exclusion. Furthermore, a post hoc Tukey honest significant difference (HSD) test was performed to identify any differences in the data.

In addition, Pearson correlation coefficient (*r*) was utilized to explore the relationship between image features and agronomical data. The study also employed a multiple linear regression to determine the added value of image features in modeling agronomical data, with the calculation of coefficient of determination *R*^2^. The hypothesis was that GF and DI were good indicators of plant health and could improve the estimation model performances.

Finally, paired *t* tests were conducted to compare BRFs and VIs obtained from the leaves with those obtained from the entire image and green elements.

## Results

### Disease pressure

**Fig. 3.**
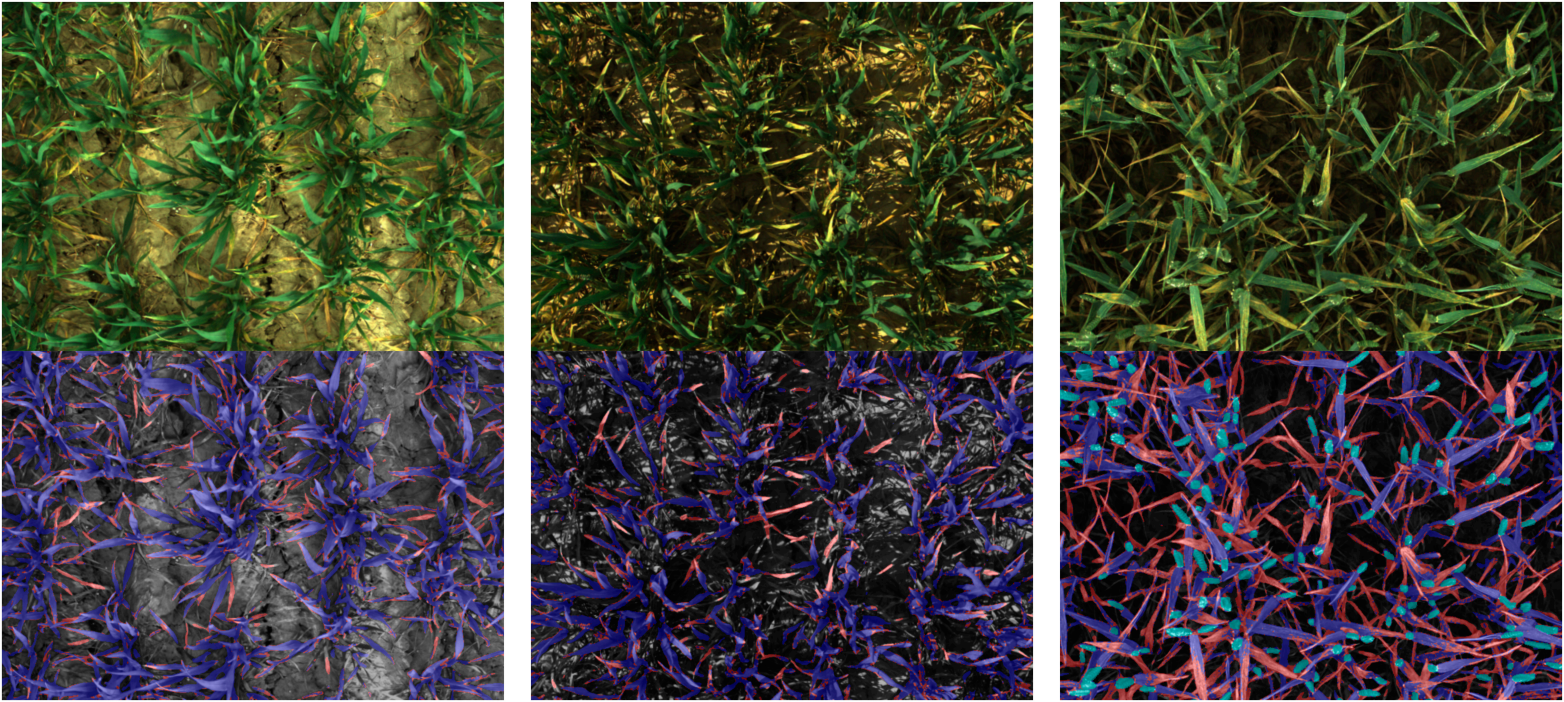
Examples of segmentation utilizing the SegVeg approach, which combines the EfficientNetB2 and XGBoost models, along with ear segmentation employing YOLOv5 and DeepMAC on April 25, May 5, and May 30, respectively, from left to right. In the segmentation results, the soil regions are depicted in shades of gray, green plants in blue, ears in sky blue, and damages in red.

The EfficientNetB2 backbone demonstrated the best performance, achieving an IoU of 0.76 and an accuracy of 0.87 for soil segmentation on the validation set (Table 3). In terms of distinguishing between green elements and damaged ones, both pixel-wise classifiers showed high accuracy and IoU. Notably, the XGBoost model was selected over the SVM due to its speed, being approximately 10 times faster. Consequently, the SegVeg model was formulated by combining the EfficientNetB2 and XGBoost models to effectively segment the entire dataset. This unified model facilitated highly accurate segmentation of soil, green elements, and damaged regions, even in difficult strong direct sunlight conditions, as visually demonstrated in Fig. 3.

When the SegVeg approach was applied throughout the entire cropping season, it became apparent that the 2021 season was characterized by a relatively low disease pressure. However, during the grain filling period, there was a slightly increased incidence of diseases, although specific data are not presented. Among the diseases affecting wheat, STB was the primary concern, with only a few treatments reaching an average severity value score (sVS) of 0.625 in early July. YR was detected on less than 20% of the plots and had a maximum average sVS of 0.625.

**Fig. 4.**
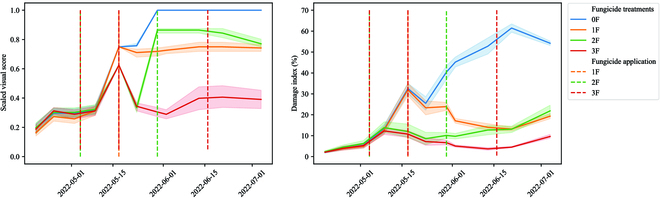
Scaled visual score and damage index curves during the 2022 season. Shaded bands represent standard deviation.

In contrast, the climatic conditions experienced in 2022 resulted in the early onset of YR at the end of April, specifically at GS 30. Subsequently, the disease exhibited significant development across all experimental plots during the stem elongation period, as depicted in Fig. 4. Notably, the 0F treatment displayed the highest sVS and DI throughout the season, with DI reaching a maximum of approximately 60%. Conversely, the 1F treatment effectively managed the disease pressure through the application of fungicide on May 17, resulting in a stabilization of sVS and a decrease in DI. The 2F and 3F treatments exhibited similar dynamics of disease pressure until the end of May, as illustrated in Fig. 4 and Table S2. However, after the flowering stage, the effectiveness and timing of application of the 3F treatment became evidently discernible.

**Fig. 5.**
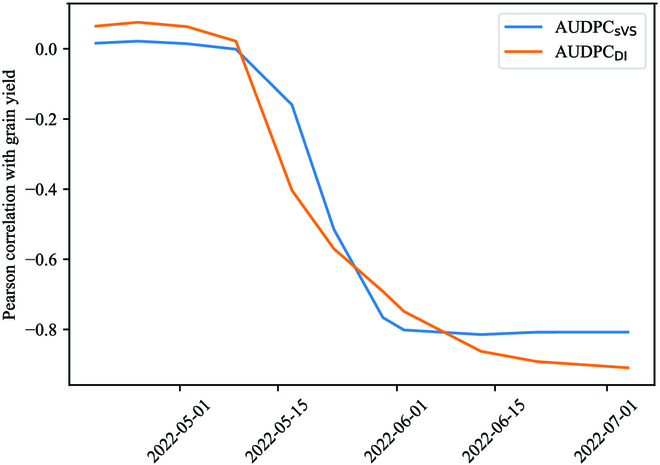
Pearson correlation between the 2022 grain yield, and both AUDPC _sVS_ and AUDPC_DI_.

The calculation of the area under the disease progress curve (AUDPC) serves as a reliable indicator of the overall disease pressure throughout the cropping season, offering an advantage over single-point notations such as sVS or DI. The correlation between AUDPC _sVS_ and AUDPC _DI_ and the final grain yield indicates that as the season progressed, the correlations became stronger (in absolute value), as depicted in Fig. 5. Notably, both AUDPC measurements exhibit a high correlation after the flowering stage. However, the proposed method, AUDPC _DI_, demonstrates a higher correlation compared to AUDPC _sVS_, which tends to reach a plateau earlier in the season, around May 30. Furthermore, prior to any fungicide applications, the AUDPC _DI_ values for the lower fertilization treatment were statistically different from those of the higher nitrogen treatments, as indicated in Table S2. In a broader sense, it can be observed that the Tukey HSD grouping was initially determined based on the nitrogen treatment at the beginning of the season and subsequently based on the fungicide treatment, which transitioned from May 9 to May 17. This observation reveals that the differences initially arose from variations in nitrogen input and subsequently from variations in disease pressure.

### Disease effects on bidirectional reflectance factor

**Fig. 6.**
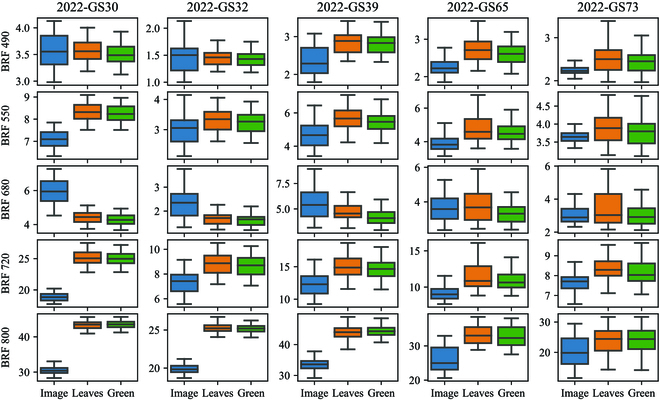
Boxplot of the bidirectional reflectance factor according to its source, i.e., from the entire image, only the leaves and only the green elements at different growth stages.

In Fig. 6, the blue boxplot represents the entire image signal, which includes soil reflectance. Comparing the blue boxplot to the boxplots of leaf and green element reflectance, significant differences were observed across most dates for all BRFs, except for the dates specified in Table S4. Furthermore, the differences in BRFs were more pronounced early in the season when the canopy cover was low. For BRF 800, the maximum variation rate, calculated as the percentage difference between the mean BRF 800 and the mean BRF 800 of leaves, reached up to 41.8% (Table 4).

The first hypothesis of this study posited that diseases or any damaging stress could impact the BRFs of the crop, primarily due to the presence of lesions and the potential signal disturbance caused by soil. The results revealed that BRFs obtained from the leaves and those derived from the green elements exhibited similar values; however, a paired *t* test revealed statistically significant differences between them, with a few exceptions (refer to Table S3). Notably, BRF 680 demonstrated higher variation compared to BRF 800, which was minimally affected (Table 4). For instance, in 2022-GS65, disease led to a reduction of 12.5% in between BRF 680 of the leaves and of the green elements.

Moreover, a strong correlation was observed between the discrepancy in BRFs derived from the leaves and those from the green elements, and DI, representing the extent of disease (see Table S5). The majority of these correlations exceeded 0.5 in absolute value, with values as high as 0.9 observed during periods of heightened disease pressure. These findings were consistent when analyzing VIs as well. It can be concluded that the discrepancy of BRFs was clearly influenced by the amount of damages in this study.

The second hypothesis of this study proposed that diseases affect not only visibly symptomatic plant parts but also symptomless ones, with varying impacts depending on the nutritional strategy of the disease [32]. Consequently, this phenomenon could potentially disrupt the measurement of green element BRFs. To exemplify this effect, NDRE was selected as a well-established VI associated with nitrogen status. ANOVA revealed that NDRE _green_, computed on healthy areas, was influenced not only by fertilization but also by the fungicide treatment (refer to Table 5).

**Fig. 7.**
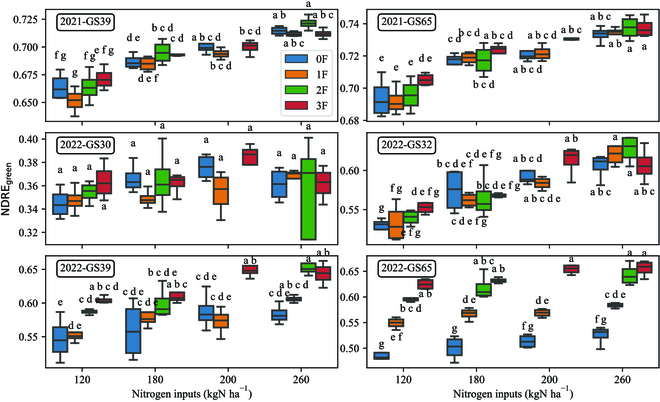
NDRE _green_ according to the treatment for major growth stages. Letters represent the groups created by the post hoc Tukey HSD test.

Furthermore, during GS39 in 2022, the NDRE _green_ value for the 120_3F treatment was found to belong to the same group as the other 3F treatments, but not with the 120_0F treatment (Fig. 7). This discrepancy indicates that the presence of disease impacted the NDRE _green_ value for the 120_3F treatment differently compared to the 120_0F treatment.

Additionally, it was observed that the difference (Δ_3F − xF_), which represents the variation between the values obtained from full protection and those obtained with reduced protection under constant nitrogen input, exhibited a strong correlation with DI for both BRFs _green_ and VIs _green_. Specifically, the correlation between Δ_3F − xF_ of NDRE _green_ and DI exceeded 0.70 starting from May 17, as indicated in Table S6.

### Analysis and modeling of nitrogen status variables under fertilization and fungicide treatments

The ANOVA revealed only one significant interaction term for all nitrogen status variables (see Table 5)—%N leaves 2021-GS65. However, values close to 0.05 were also observed for 2022-GS65 for NNI and %N leaves. Therefore, while the 2 factors can be analyzed separately, caution must be exercised when drawing conclusions.

The fertilization factor significantly impacted most variables, with %N leaves and %N total showing significance at tillering (2022-GS30). However, no effect was observed on Nuptake leaves and Nuptake total for 2021-GS39, as well as on NNI, Nuptake total, and %N total for 2022-GS75.

Fungicide did not affect NNI and %N total, except for the maturity GS. However, Nuptake leaves and Nuptake total were significantly influenced by fungicide starting from 2022-GS65. %N leaves were affected earlier, at 2022-GS39.

**Fig. 8.**
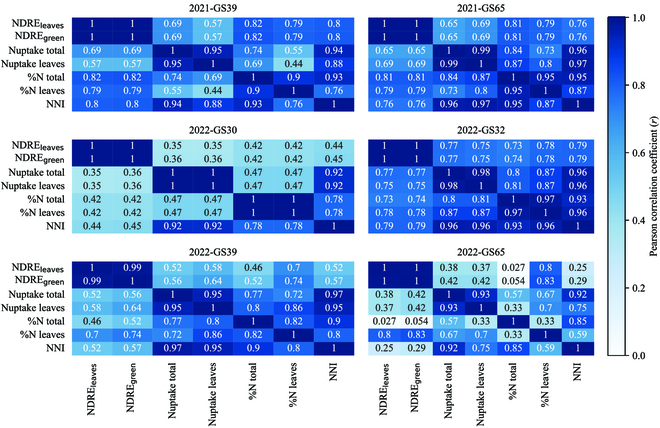
Correlation matrix between NDRE and nitrogen status variables at different growth stages.

NDRE_green_ displayed a similar trend to %N leaves in 2022 regarding the impact of fungicide application. However, it was not affected by fertilization at GS75. In 2021, it was influenced by both fertilization and fungicide. Additionally, NDRE_green_ exhibited a stronger correlation with leaf nitrogen status than NDRE_leaves_ when disease was present, specifically from GS 39 to 75 in 2022 (Fig. 8) . Meanwhile, the correlation with NNI was much lower in 2022 compared to 2021 for GS 39 and 65. It is noteworthy that there was a decrease in the correlation between %N of leaves and plant nitrogen status variables, such as NNI and %N total, during periods of high disease pressure. Moreover, an interesting result is that the correlation between NDRE_leaves_ and NDRE_green_ was perfect, while the values may differ (see the above section).

Finally, some multiple regression analyses were conducted using features from both RGB and multispectral imagery. The inclusion of GF and DI improved most of the model performances for dates with high disease pressure (Table S7). Moreover, it also improve general model that encompasses all data, i.e., dates and plots.

## Discussion and Conclusion

### CNN as a promising damage detection tool

Plant diseases are commonly identified through the observation of visible symptoms, a process conducted by agronomists to assess the plant’s resistance capabilities. However, this approach can be time-consuming, labor-intensive, and susceptible to subjectivity. To address these limitations, a scoring method named SegVeg was proposed by Serouart et al. [39] for evaluating nongreen elements, predominantly characterized as disease symptoms in the present study. The SegVeg model employed a 2-step methodology that involved generating a mask with 3 distinct categories: soil, green plant parts, and damaged parts. This approach utilized a U-NET architecture in conjunction with a pixel-wise classifier. While the model yielded satisfactory segmentation masks, it was found that misclassified pixels could arise under direct sunlight conditions [39]. To mitigate this issue, additional data encompassing various illumination conditions were incorporated into the VegAnn dataset as part of the current study. Nevertheless, due to the inherent scattered nature of the wheat canopy, particularly in intense sunlight conditions, the creation of accurate masks became more challenging, as exemplified in the left image of Fig. 3. Specifically, the lower regions of the canopy tended to exhibit significant darkness. The utilization of a high dynamic range (HDR) camera presents a potential solution to alleviate the aforementioned issues.

Both visual scoring and the SegVeg method have demonstrated their efficacy in characterizing the impact of fungicide treatment on wheat plants. In contrast to the study conducted by Koc et al. [57], these 2 methods are distinct and yield different outputs. However, they can be utilized for similar purposes, such as estimating the effect of disease on yield loss. The SegVeg method lead to DI that objectively quantifies the extent of damage in a nadir view, providing a 2D assessment. On the other hand, visual scoring considers the disease intensity on the most significant leaves. In our study, DI indicated very high levels of infestation, exceeding 50% in the zero protection treatment, which resulted in a completely devastated plot even under natural inoculation. Conversely, the well-protected treatment, with minimal disease observed by human assessors, yielded a DI value of 10%, suggesting a slight overestimation by the models. The current nadir view system restricts observations to visible symptoms on the upper leaves, limiting its capacity to assess diseases such as STB that primarily develop in the lower canopy. To address this limitation, a potential solution could involve implementing a system that opens the canopy, similar to the manual manipulation performed by human assessors during visual scoring. This approach could be combined with an object detection CNN to detect and quantify disease spots, as demonstrated by Schirrmann et al. [58]. Furthermore, extending the pixel-wise annotation to include damage classification could offer enhanced insights into the nature of the damage. However, this would require substantial efforts in image acquisition and annotation tasks.

The nadir view perspective can also explain the observed decrease in DI when new green leaves emerge. This phenomenon led to the utilization of the AUDPC as a metric to account for the negative impact of disease throughout the season, which proves to be a suitable measure for studying its influence on grain yield. As the season progresses, the treatments became more distinguishable from one another, and their correlation with the final grain yield has strengthened. Similar findings were reported by Zhou et al. [59] using GF. Toward the end of the season, computer vision techniques surpassed the visual scoring method, likely because sVS reaches its maximum value early on and can no longer differentiate between the different treatments. Notably, foliar diseases have a detrimental effect on carbon accumulation by reducing the green leaf area until senescence occurs [33,32]. However, the fungicide mixture, consisting of triazoles-pyrazoles-carboxamides, may also significantly impact the green leaf area, while the last triazole appeared to have no effect. This fungicide interaction adds complexity to the already complex relationship between nitrogen plant fluxes and rust severity [60]. In fact, biotrophic pathogens like YR usually benefit from high nitrogen availability [32]. However, in this study, no statistically significant differences were observed in DI to confirm this statement.

Last, it is worth noting that the grouping of AUDPC_DI_ in Table S2 initially focused on nitrogen input and subsequently on fungicide treatment. This suggests that distinguishing between different nitrogen treatments may not be possible beyond a certain level of disease pressure, as the plant is unable to fully recover from the damage.

### Disease affects reflectance in 2 ways

The findings of this study highlight the importance of accurately differentiating the elements present in multispectral imagery of crops. Specifically, the removal of soil and other background elements from crop scenes is crucial for the proper evaluation of crop phenotypes, as shown in Table 4.

Similar conclusions can be drawn regarding the impact of diseases. Diseases, through their symptoms, lead to a reduction in healthy areas [33]. The results of the study indicate that damage symptoms have a significant effect on BRFs, with the 680-nm wavelength exhibiting a particularly pronounced impact. This suggests that spectral data at this specific wavelength can serve as a valuable tool for distinguishing between healthy and diseased plants [22,61,62]. Furthermore, a strong correlation was observed between variations in BRFs and the extent of damage caused by the disease, indicating that as the disease progresses, the differences in BRFs become more pronounced.

The study also revealed that diseases affect the BRFs of the green area of the plant. It was hypothesized that diseases can significantly impact the biophysical and biochemical properties of wheat plants, thereby influencing the measurement of BRFs and subsequent VIs [32]. In the presence of disease, the measurement of NDRE _green_ showed a stronger correlation with nitrogen status variables compared to NDRE _leaves_. Specifically, it exhibited a high correlation with leaf nitrogen concentration and leaf nitrogen uptake, but not with other nitrogen status parameters. However, the nitrogen status of the leaves appeared also to deviate from other nitrogen parameters such as the NNI and the overall plant nitrogen concentration. This deviation is likely due to the complex influence of diseases on the overall nitrogen status of the plants [32]. In fact, YR was found to impact the photosynthetic capacity of the green elements, but not the nitrogen content of the stem [33]. Therefore, while spectral measurements, which primarily capture information from leaves, may effectively represent the nitrogen status of the leaves, they may not necessarily reflect the nitrogen status of the entire plant. This has important implications for fertilization decision-making tools. It was observed that a single value of NDRE_green_ could represent different nitrogen input levels (Fig. 7), which could potentially result in misleading interpretations. This is particularly relevant when considering the last fertilization input made at GS 39 in Belgium.

To address this issue, it is important to carefully consider disease quantification in nitrogen estimation models based on spectral data. The use of NDRE_green_, or the addition of features from the RGB image, such as DI, could aid in modeling nitrogen status variables. In addition, on each individual date, we observed a strong and significant correlation between both NDRE values. It is important to clarify that this correlation does not imply that the values are identical, but rather indicates a high degree of association between them. Consequently, using these NDRE measurements in relative terms may not pose any issues. However, in the context of a larger case study or when considering absolute values, it is possible that discrepancies or challenges may arise. In fact, it can be challenging to develop a model that accurately represents the nitrogen concentration for all GSs, while the total nitrogen uptake is more easily assessed across all dates [63]. Hyperspectral systems have an advantage in distinguishing between nitrogen deficiency and rust infection, as they use narrower wavebands [34]. Additionally, from a nadir view, they are more effective at detecting diseases that develop in the canopy by sensing overall plant health [64]. However, their use in the field may be limited due to practical constraints, higher costs, and equipment complexity

Last, during the research, an unsupervised clustering model was tested using all image features. Initially, the model clustered the plots based on nitrogen levels, and subsequently, from the middle of May, it differentiated them according to fungicide treatment, even when utilizing RGB features (data not shown). This further supports our earlier assertion that above a certain threshold of disease pressure, accurately determining the nitrogen treatment of a plot without historical information becomes challenging. It became evident that studying temporal features emerged as a reliable approach for disentangling stress factors [19,22]. Therefore, based on these findings, it is recommended to conduct further research on nitrogen stress modeling using spectral data in the presence of disease. It is worth noting that different diseases may exhibit distinct interactions with plant nitrogen status, as explained by Simón et al. [32], implying that the observations made in this study for YR may not necessarily apply to other diseases such as Septoria.

## Data Availability

The data presented in this study are available on request from the corresponding author.
