# Supplementary material for: To What Extent Does Yellow Rust Infestation Affect Remotely Sensed Nitrogen Status?
Source: Plant Phenomics. 2023 Sep 6;5:0083. doi: 10.34133/plantphenomics.0083 (PMC10482323; doi:10.34133/plantphenomics.0083)
Supplement: Supplementary 1 — Tables S1 to S7 [file plantphenomics.0083.f1.pdf]

Supplementary Materials

Table S1: Vegetation indices selected in this study

Name	VI forumlation	Reference
NDRE	$\frac{\lambda 800-\lambda 720}{\lambda 800+\lambda 720}$	[51]
mNDb	$\frac{\lambda 490-\lambda 720}{\lambda 490+\lambda 800}$	[18]
Cirede	$\frac{\lambda 800}{\lambda 720}-1$	[52]
NDVI	$\frac{\lambda 800-\lambda 680}{\lambda 800+\lambda 680}$	[53]
PSRI	$\frac{\lambda 680-\lambda 490}{\lambda 800}$	[54, 55]

Table S2: Tukey HSD test to investigate the effect of the treatment on the $AUDPC_{DI}$ across the cropping season.

Treatment	19/04/2022	25/04/2022	02/05/2022	09/05/2022	17/05/2022	23/05/2022	30/05/2022	02/06/2022	13/06/2022	21/06/2022	04/07/2022
120_0F	a b	a b c	a b c d	a	a	a b	a b	a b	a b c	a	a
120_1F	a b	a b	a b c	a	a	a b	a b c d	a b c d	c d e	b c	b
120_2F	a	a	a	a	a	a b	a b c d	b c d e	d e f g	b c d	b c
120_3F	a b	a b	a b	a	a	a b	b c d	c d e	e f g	c d	c
180_0F	b c	a b c d	a b c d	a	a	a b	a b	a	a	a	a
180_1F	a b c	a b c d	a b c d	a	a	a b	a b c d	a b c d e	c d e f	b c	b
180_2F	b c	a b c d	a b c d	a	a	a b	b c d	c d e	d e f g	b c d	b c
180_3F	a b c	a b c d	a b c d	a	a	a b	c d	d e	f g	d	c
200_0F	c	b c d	a b c d	a	a	a	a	a	a	a	a
200_1F	b c	b c d	a b c d	a	a	a b	a b c d	a b c d e	c d e	b	b
200_3F	c	c d	b c d	a	a	a b	c d	d e	f g	d	c
260_0F	c	d	c d	a	a	a b	a b	a b	a b	a	a
260_1F	c	d	d	a	a	a b	a b c	a b c	b c d	b	b
260_2F	c	c d	b c d	a	a	a b	b c d	c d e	d e f g	b c d	b c
260_3F	c	d	b c d	a	a	b	d	e	g	d	c

Table S3: Comparison of variable between the whole leaf and the green leaf, using a paired t-test. The p-value indicates the level of statistical significance of the difference between the two groups.

	BRF_490	BRF_550	BRF_680	BRF_720	BRF_800	BRF_900	NDVI	NDRE	PSRI	mNDb	CIrede
02/06/2021	<0.01	<0.01	<0.01	<0.01	<0.01	<0.01	<0.01	<0.01	<0.01	<0.01	<0.01
16/06/2021	<0.01	<0.01	<0.01	<0.01	<0.01	<0.01	<0.01	<0.01	<0.01	<0.01	<0.01
17/03/2022	<0.01	<0.01	<0.01	<0.01	<0.01	<0.01	<0.01	<0.01	<0.01	0.457	<0.01
11/04/2022	<0.01	<0.01	<0.01	<0.01	<0.01	<0.01	<0.01	<0.01	<0.01	<0.01	<0.01
19/04/2022	<0.01	<0.01	<0.01	<0.01	<0.01	<0.01	<0.01	<0.01	<0.01	<0.01	<0.01
25/04/2022	<0.01	<0.01	<0.01	<0.01	<0.01	<0.01	<0.01	<0.01	<0.01	<0.01	<0.01
02/05/2022	<0.01	<0.01	<0.01	<0.01	<0.01	<0.01	<0.01	<0.01	<0.01	<0.01	<0.01
09/05/2022	<0.01	<0.01	<0.01	<0.01	<0.01	<0.05	<0.01	<0.01	<0.01	<0.01	<0.01
17/05/2022	<0.01	<0.01	<0.01	<0.01	<0.01	<0.01	<0.01	<0.01	<0.01	<0.01	<0.01
23/05/2022	<0.05	0.083	<0.01	<0.05	<0.01	<0.01	<0.01	<0.01	<0.01	<0.01	<0.01
30/05/2022	<0.01	0.063	<0.01	<0.01	<0.01	<0.01	<0.01	<0.01	<0.01	<0.01	<0.01
02/06/2022	<0.01	<0.01	<0.01	<0.01	<0.01	<0.01	<0.01	<0.01	<0.01	<0.01	<0.01
13/06/2022	<0.01	<0.01	<0.01	<0.01	0.825	0.309	<0.01	<0.01	<0.01	<0.01	<0.01
21/06/2022	<0.01	<0.01	<0.01	<0.01	<0.01	0.573	<0.01	<0.01	<0.01	<0.01	<0.01
04/07/2022	<0.01	<0.01	<0.01	<0.01	<0.01	<0.01	<0.01	<0.01	<0.01	<0.01	<0.01

Table S4: Comparison of variable between the whole leaf and the image, using a paired t-test. The p-value indicates the level of statistical significance of the difference between the two groups.

	BRF_490	BRF_550	BRF_680	BRF_720	BRF_800	BRF_900	NDVI	NDRE	PSRI	mNDb	CIrede
02/06/2021	<0.01	<0.01	<0.01	<0.01	<0.01	<0.01	<0.01	<0.01	<0.01	<0.01	<0.01
16/06/2021	<0.01	<0.01	<0.01	<0.01	<0.01	<0.01	<0.01	<0.01	<0.01	<0.01	<0.01
17/03/2022	<0.01	<0.01	<0.01	<0.01	<0.01	<0.01	<0.01	<0.01	<0.01	<0.01	<0.01
11/04/2022	0.371	<0.01	<0.01	<0.01	<0.01	<0.01	<0.01	<0.01	<0.01	<0.01	<0.01
19/04/2022	<0.01	<0.01	<0.01	<0.01	<0.01	<0.01	<0.01	<0.01	<0.01	<0.01	<0.01
25/04/2022	<0.01	<0.05	<0.01	<0.01	<0.01	<0.01	<0.01	<0.01	<0.01	<0.01	<0.01
02/05/2022	0.380	<0.01	<0.01	<0.01	<0.01	<0.01	<0.01	<0.01	<0.01	<0.01	<0.01
09/05/2022	<0.01	<0.01	<0.01	<0.01	<0.01	<0.01	<0.01	<0.01	<0.01	<0.01	<0.01
17/05/2022	<0.01	<0.01	<0.01	<0.01	<0.01	<0.01	<0.01	<0.01	<0.01	<0.01	<0.01
23/05/2022	<0.01	<0.01	<0.01	<0.01	<0.01	<0.01	<0.01	<0.01	<0.01	<0.01	<0.01
30/05/2022	<0.01	<0.01	<0.01	<0.01	<0.01	<0.01	<0.01	<0.01	<0.01	0.142	<0.01
02/06/2022	<0.01	<0.01	<0.01	<0.01	<0.01	<0.01	<0.01	<0.01	<0.01	0.843	<0.01
13/06/2022	<0.01	<0.01	<0.01	<0.01	<0.01	<0.01	<0.01	<0.01	<0.01	<0.01	<0.01
21/06/2022	<0.01	<0.01	<0.01	<0.01	<0.01	<0.01	<0.01	<0.01	<0.01	<0.01	<0.01
04/07/2022	<0.01	<0.01	<0.01	<0.01	<0.01	<0.01	<0.01	<0.01	<0.01	<0.01	<0.01

Table S5: Pearson's correlation between $\Delta_{\text{leaves-green}}$ and the damage index.

	BRF_490	BRF_550	BRF_680	BRF_720	BRF_800	BRF_900	NDVI	NDRE	PSRI	mNDb	CIrede
02/06/2021	-0.55	-0.52	0.02	-0.84	-0.95	-0.95	-0.55	-0.62	0.48	-0.63	-0.46
16/06/2021	0.19	0.13	0.33	-0.05	-0.58	-0.62	-0.71	-0.51	0.54	-0.36	-0.11
17/03/2022	0.63	0.35	0.74	-0.07	-0.68	-0.66	-0.77	-0.78	0.80	0.40	-0.70
11/04/2022	0.59	0.29	0.64	0.35	-0.04	-0.01	-0.76	-0.72	0.69	-0.56	-0.57
19/04/2022	0.88	0.92	0.97	0.85	-0.68	-0.62	-0.98	-0.97	0.98	-0.97	-0.93
25/04/2022	0.44	0.58	0.89	-0.42	-0.81	-0.80	-0.91	-0.89	0.92	-0.81	-0.81
02/05/2022	0.88	0.96	0.96	0.95	0.67	0.74	-0.95	-0.95	0.95	-0.97	-0.76
09/05/2022	0.17	0.67	0.81	0.60	-0.40	-0.21	-0.84	-0.70	0.89	-0.75	-0.53
17/05/2022	0.45	0.73	0.92	0.69	-0.27	-0.23	-0.93	-0.92	0.93	-0.93	-0.84
23/05/2022	-0.46	-0.39	0.80	-0.36	-0.78	-0.74	-0.96	-0.64	0.98	-0.65	-0.58
30/05/2022	-0.76	0.21	0.98	0.04	-0.71	-0.71	-0.98	-0.91	0.99	-0.88	-0.88
02/06/2022	0.94	0.96	0.98	0.95	0.87	0.87	-0.99	-0.98	0.99	-0.97	-0.94
13/06/2022	0.89	0.91	0.97	0.90	0.73	0.79	-0.99	-0.96	0.99	-0.96	-0.86
21/06/2022	0.91	0.90	0.98	0.89	0.47	0.66	-0.96	-0.93	0.96	-0.90	-0.81
04/07/2022	0.97	0.90	0.93	0.93	0.92	0.93	-0.78	-0.81	0.89	-0.45	-0.17

Table S6: Pearson's correlation between $\Delta_{3F\text{-}xF}$ and the damage index.

	BRF_490_{green}	BRF_550_{green}	BRF_680_{green}	BRF_720_{green}	BRF_800_{green}	BRF_900_{green}	$NDVI_{green}$	$NDRE_{green}$	$PSRI_{green}$	$mNDb_{green}$	$Cirede_{green}$
02/06/2021	-0.04	-0.20	-0.15	-0.04	0.53	0.49	0.60	0.70	-0.26	0.65	0.67
16/06/2021	0.00	-0.24	-0.24	-0.22	0.43	0.33	0.47	0.67	-0.41	0.73	0.60
17/03/2022	-0.16	-0.15	-0.16	-0.15	-0.07	-0.03	0.21	0.31	-0.14	-0.04	0.35
11/04/2022	-0.16	-0.23	-0.18	-0.30	0.03	0.00	0.24	0.53	-0.50	0.33	0.60
19/04/2022	-0.40	-0.48	-0.47	-0.47	0.03	0.06	0.52	0.54	-0.36	0.52	0.49
25/04/2022	-0.22	-0.28	-0.31	-0.18	0.20	0.17	0.32	0.34	-0.29	0.34	0.34
02/05/2022	-0.28	-0.36	-0.45	-0.39	-0.05	-0.02	0.45	0.40	-0.65	0.42	0.38
09/05/2022	-0.35	-0.29	-0.27	-0.27	-0.21	-0.06	0.23	0.27	-0.03	0.20	0.28
17/05/2022	-0.37	-0.59	-0.58	-0.61	0.54	0.31	0.63	0.70	-0.66	0.73	0.72
23/05/2022	-0.56	-0.43	-0.59	-0.30	0.44	0.33	0.89	0.78	-0.77	0.66	0.79
30/05/2022	-0.85	-0.94	-0.93	-0.95	0.63	0.53	0.93	0.96	-0.96	0.97	0.95
02/06/2022	-0.57	-0.83	-0.83	-0.88	0.85	0.82	0.87	0.93	-0.95	0.96	0.90
13/06/2022	-0.59	-0.73	-0.88	-0.60	0.88	0.89	0.96	0.94	-0.98	0.95	0.90
21/06/2022	-0.35	-0.36	-0.45	-0.25	0.71	0.78	0.90	0.90	-0.83	0.86	0.91
04/07/2022	0.62	0.82	-0.78	0.90	0.95	0.95	0.98	0.92	-0.98	0.67	0.87

Table S7: R^2 of the linear regression associating several feature combinations with agronomic data. GF stands for green fraction and DI for damage index.

Date	Features	Nuptake total	Nuptake leaves	Nuptake stem	Nuptake Ears	%N total	%N leaves	%N stem	%N Ears	NNI
2021-GS39	$NDRE_{leaves}$	0.49	0.33	0.52		0.65	0.62	0.62		0.65
	$NDRE_{green}$	0.48	0.32	0.50		0.67	0.63	0.64		0.65
	$NDRE_{green}$ + GR	0.53	0.34	0.62		0.67	0.64	0.64		0.66
	$NDRE_{green}$ + GF + DI	0.53	0.34	0.62		0.68	0.64	0.64		0.66
2021-GS65	$NDRE_{leaves}$	0.43	0.49	0.46	0.05	0.66	0.63	0.62	0.38	0.58
	$NDRE_{green}$	0.42	0.47	0.45	0.05	0.66	0.63	0.61	0.37	0.57
	$NDRE_{green}$ + GR	0.46	0.50	0.48	0.15	0.66	0.68	0.61	0.37	0.58
	$NDRE_{green}$ + GF + DI	0.46	0.50	0.48	0.15	0.67	0.68	0.62	0.39	0.58
2022-GS30	$NDRE_{leaves}$	0.13	0.13			0.17	0.17			0.20
	$NDRE_{green}$	0.13	0.13			0.17	0.17			0.20
	$NDRE_{green}$ + GR	0.20	0.20			0.17	0.17			0.23
	$NDRE_{green}$ + GF + DI	0.21	0.21			0.42	0.42			0.32
2022-GS32	$NDRE_{leaves}$	0.61	0.58	0.52		0.53	0.59	0.33		0.63
	$NDRE_{green}$	0.59	0.57	0.49		0.54	0.61	0.33		0.62
	$NDRE_{green}$ + GR	0.59	0.57	0.50		0.54	0.62	0.33		0.62
	$NDRE_{green}$ + GR+ DI	0.59	0.57	0.50		0.59	0.62	0.47		0.63
2022-GS39	$NDRE_{leaves}$	0.28	0.38	0.08		0.27	0.50	0.01		0.30
	$NDRE_{green}$	0.32	0.41	0.11		0.27	0.55	0.01		0.33
	$NDRE_{green}$ + GR	0.34	0.42	0.15		0.28	0.56	0.02		0.35
	$NDRE_{green}$ + GF + DI	0.38	0.52	0.15		0.39	0.64	0.02		0.43
2022-GS65	$NDRE_{leaves}$	0.18	0.19	0.03	0.32	0.00	0.69	0.03	0.01	0.08
	$NDRE_{green}$	0.18	0.18	0.03	0.35	0.00	0.69	0.03	0.02	0.08
	$NDRE_{green}$ + GR	0.18	0.23	0.03	0.35	0.04	0.69	0.06	0.22	0.09
	$NDRE_{green}$ + GR+ DI	0.47	0.48	0.28	0.40	0.50	0.85	0.29	0.35	0.54
2022-GS73	$NDRE_{leaves}$	0.57	0.50	0.06	0.43	0.02	0.48	0.29	0.08	0.29
	$NDRE_{green}$	0.59	0.51	0.06	0.46	0.02	0.49	0.30	0.09	0.31
	$NDRE_{green}$ + GR	0.60	0.54	0.06	0.49	0.06	0.53	0.30	0.17	0.34
	$NDRE_{green}$ + GF + DI	0.67	0.70	0.14	0.51	0.11	0.57	0.32	0.18	0.41
All	$NDRE_{leaves}$	0.54	0.64	0.41	0.01	0.39	0.01	0.00	0.17	0.46
	$NDRE_{green}$	0.58	0.61	0.43	0.00	0.44	0.03	0.00	0.16	0.46
	$NDRE_{green}$ + GR	0.59	0.63	0.44	0.16	0.56	0.35	0.05	0.35	0.47
	$NDRE_{green}$ + GF + DI	0.64	0.63	0.59	0.40	0.72	0.50	0.10	0.35	0.48
